# Mortality among Canadian population with multimorbidity: A retrospective cohort study

**DOI:** 10.1177/26335565231157626

**Published:** 2023-02-17

**Authors:** Xiang Xiao, Jeremy Beach, Ambikaipakan Senthilselvan

**Affiliations:** 1 School of Public Health, 3158 University of Alberta, Edmonton, Alberta, Canada; 2 Department of Medicine, 3158 University of Alberta, Edmonton, Alberta, Canada

**Keywords:** Adults, chronic diseases, multimorbidity, mortality

## Abstract

**Objective:**

The aim of this study was to examine the effect of multimorbidity and the joint effect of chronic diseases on all-cause mortality among subjects aged 35 years and above.

**Study Design:**

Population-based retrospective cohort study.

**Methods:**

Multimorbidity was defined by the respondent’s self-report of having two or more chronic diseases of the nine considered. The Canadian Community Health Surveys conducted in 2003/2004, 2005/2006 and 2007 to 2014 were linked with the Canadian Vital Statistics Death Database to examine the association between multimorbidity and all-cause mortality in subjects aged 35 years and above. Cox’s proportional hazards models were used to estimate risk of multimorbidity on death after adjusting for the confounders in three age groups.

**Results:**

Multimorbidity had an increased risk of death in all three age groups with the youngest having the highest risk after adjusting for potential confounders (35 to 54 years: hazard ratio (HR) = 3.77, 95% CI: 3.04, 4.67; 55 to 64 years: HR = 2.64, 95% CI: 2.36, 2.95; 65 years and above: HR = 1.71; 95% CI:1.63,1.80). Subjects with cancer had the highest risk of death in the three age groups. When the interactions between chronic diseases were considered, subjects with COPD and diabetes had a significantly increased risk of death in comparison to those without COPD or diabetes in the 55 to 64 years. (HR = 2.59, 95% CI: 2.01, 3.34).

**Conclusions:**

Prevention of multimorbidity should be targeted not only in the older population but also in the younger populations. Synergistic effects of chronic diseases should be considered in the management of multimorbidities.

## Introduction

The increasing life expectancy and prevalence of multimorbidity in Canada and worldwide has attracted public health attention during recent years.^1^ Studies have demonstrated that multimorbidity is more prevalent in older adults.^2,3^ It has also been reported that multimorbidity is associated with a greater risk of disability, frailty and reduction in quality of life with consequent costs to society as a whole.^4,5^ A longitudinal study based on the National Health and Nutrition Examination Survey (NHANES) from the USA reported that participants had a 23% increased risk of death for each additional morbidity after adjusting for potential confounders (HR = 1.23; 95% CI: 1.19, 1.28; *p* < 0.001).^6^ A Canadian longitudinal study conducted in Ontario reported that people with multimorbidity had a higher risk of death than those, without which was independent of their age.^7^ However, increased risk of death associated with multimorbidity was not observed in some studies.^8,9^ Multimorbidity in the elderly population appears to be multifactorial and is associated with sociodemographic characteristics,^10^ health behavior^6^ and frailty.^11^ In a population-based cohort study from Denmark, subjects with lower levels of education had a greater risk of mortality in comparison to those with higher levels of education.^12^ Studies have also reported that the risk of mortality associated with multimorbidity decreased with ageing.^13^

Although most of the published studies have assumed that all comorbidities have similar risk associated for mortality, it is likely that certain comorbidities have a greater risk of mortality than others. For example, it has been reported that COPD patients with diabetes have a higher risk of mortality in comparison to COPD patients without diabetes.^14^ Subjects with asthma and mental health problems were reported to have an increased risk of death in comparison to those who had only asthma.^15^ Thus, considering the most prevalent or harmful combination of comorbidities rather than treating them individually may allow health professionals to intervene and prevent death. However, studies on multimorbidity and its effect on mortality are limited and only a few studies have examined the joint effect of chronic diseases on mortality.^16,17^

The primary objective of this study was to examine the relationship between multimorbidity and all-cause mortality in the Canadian population. The secondary objective was to examine the independent and interaction effects of chronic diseases on mortality. These objectives were examined in a population-based retrospective cohort study by linking Canadian Community Health Survey (CCHS) data with Canadian Vital Statistics Death (CVSD) data.

## Methods

### Data Source

The Canadian Community Health Survey (CCHS) is a cross-sectional survey including the population aged 12 years and above administrated by Statistics Canada.^18^ The information collected in CCHS included demographic characteristics, health status, health care utilization, and health determinates. The CCHS target population includes people living in ten provinces and three territories, excluding those living on reserves and aboriginal settlements, those full-time in the Canadian forces, the institutionalized population, 12-17 years old children living in foster care, and persons living in the health regions of Région du Nunavik and Région des Terres-Cries-la-Baie-James in Quebec. A multi-stage sampling allocation strategy was used to provide a representative sampling distribution to the health regions and the provinces. The first CCHS was conducted in 2000/2001 (Cycle 1.1) and was followed by Cycle 2.1 in 2003/2004, and Cycle 3.1 in 2005/2006 with a total sample size of approximately 13,000. Starting in 2007, the sample size was increased to approximately 65,000 in each cycle and conducted annually instead of every two years. The data from the ten CCHS cycles (2003/2004, 2005/2006, 2007-2014) were considered in this study. As the age-standardized mortality rates of the participants in CCHS conducted in 2000/2001 was lower than the other CCHS years, the data from this survey were excluded from this study because of the potential to introduce healthy respondent bias.^19^

The Canadian Vital Statistics Death Database (CVSD) included information of all deaths registered in Canada annually and monthly from 1921 onwards.^20^ Reports of deaths were submitted to Statistics Canada through the province and territorial vital statistics registries. The information related to each death included age, sex, marital status, place of residence, birthplace of the deceased, date of death, cause of death as classified by International Classification of Diseases (ICD) (using the version in effect at the time of death), province or territory of occurrence of death, place of accident (for most non-transport accidental deaths) and results of autopsy if one was held. If the autopsy was held, the results of the autopsy were taken into account in establishing the cause of death.^20^

### Data linkage

The linkage of CCHS to CVSD was given approval by the Chief Statistician of Canada.^21^ The subjects from CCHS (2003/2004, 2005/2006, 2007-2014) who granted permission to share and link their survey answers were included in the linkage process. The cohort files were created by a probabilistic linkage of CCHS subjects to Derived Record Depository (DRD) in the Social Data Linkage Environment (SDLE) to enhance data quality. In the interest of creating more accurate results, probabilistic record linkage employed several non-unique identifiers, such as name, sex, date of birth, and postal code, to determine the likelihood of records referring to the same individual.^19^ An adjustment was made to account for records that were not captured in the DRD and those who did not provide permission for their survey answers to be shared and linked.^22^ The same linkage methodology was used with CVSD (January 1, 2000 to December 31, 2017) to create an analytical file.

### Study sample

A linking key (Stc_id) in the cohort files (CCHS survey files) and analytical file (CVSD file) was used for the linkage. In total, 629,835 (80.82%) CCHS subjects were successfully linked to death records held with CVSD from January 2003 to December 2017. Twenty individuals were excluded due to logical inconsistency (i.e., death date prior to interview date), and 194,925 CCHS-CVSD subjects aged less than 35 years old were also excluded from the study, which resulted in 434,885 subjects for the final analysis with a maximum follow-up of 14 years. The details of exclusion and inclusion during the follow up are shown in the flow chart (Figure 3.1). In total, 64,725 respondents died between the year of interview, and the end of follow-up (December 31, 2017) and 370,160 respondents were censored.

### Outcomes

The outcomes for this study were death during the period of follow-up or remaining alive at the end of follow-up (December 31, 2017), and the time to death or end of follow-up. The survival time was determined from the CCHS interview date (baseline) to the date of death or end of follow-up (censored).

### Main exposure variable

The main exposure variable was multimorbidity which was defined from the responses to the question which had an opening statement *“Now I’d like to ask about certain chronic health conditions which you may have. We are interested in "long-term conditions" which are expected to last or have already lasted 6 months or more and that have been diagnosed by a health professional. Do you have <conditions>?”* Wordings of the questions remained similar in all the CCHS cycles considered in this study. Instead of including all the chronic illnesses, this study focused on nine chronic diseases that have a high prevalence and a significant effect on health care utilization. The nine chronic diseases were asthma, arthritis, cancer, hypertension, diabetes, heart disease, stroke, mood disorder, and COPD. These chronic diseases have been previously considered in several studies on multimorbidity and are the most prevalent diseases in Canada.^23–25^ Multimorbidity was defined by the respondent’s self-report of having two or more chronic diseases.^25^ The respondents who did not report any of the nine selected chronic diseases were defined as those “without multimorbidity”. A dichotomous variable was defined to indicate multimorbidity with “1” indicating presence of multimorbidity and “0” indicating absence of morbidity. The respondents who reported a single chronic disease were excluded in this study.

### Covariates

Socio-demographic characteristics and health behaviors considered in this study were identified based on existing literature focusing on multimorbidity and mortality and were obtained from each cycle of CCHS. These factors were considered as potential confounders for the association between multimorbidity and mortality in this study. All the baseline factors were categorized as follows in the analysis: age (35-49, 50-64, 65 years and above), sex (female or male), cultural background (white, non-white), body weight (underweight, normal weight, overweight, obese), marital status (single, widowed/divorced/separated, common-law/married), province of residence (Births Columbia, Prairies, Ontario, Quebec, Atlantic, Territories), household income (less than $39,999, $40,000 to $59,000, $60,000 to $79,999, $80,000 or more), highest household education (less than secondary school graduation education, secondary school graduation no post-secondary, some post-secondary education, and post-secondary and above), smoking status (non-smoker, smoker at time of CCHS interview), alcohol (no, moderate, heavy), physical activity (inactive, moderate, active), stress (not and not very stressful, somewhat, extreme) and year of entry into the cohort.

### Statistical analysis

For each CCHS cycle, the follow up was described using the frequency and proportion of deaths, mean and total follow-up time. The association of multimorbidity and the nine individual chronic diseases with mortality was described using the unadjusted hazard ratios with 95% confidence intervals using Cox’s proportional hazards models. The unadjusted hazard ratio of multimorbidity was also obtained for each CCHS cycle separately to examine the consistency of association of multimorbidity with mortality between CCHS cycles. A Cox’s proportional hazard regression was used to determine the adjusted hazard ratio of multimorbidity after controlling for the baseline factors. Smoothed Kaplan-Meier curves were used to describe the survival distribution of subjects with and without multimorbidity in the three age groups (35 to 49, 50 to 64 and 65 years and above). The distribution of incidence of death for the categories of the baseline factors were obtained and an unadjusted hazard ratio was used to describe the strength of the association between the baseline factors and mortality for the whole sample and three age groups. Hazard ratios for multimorbidity were obtained for the three age groups after adjusting for the significant baseline factors. The assumptions of the proportional hazards model were validated visually by plots of log[log(survival time)] vs. log(time). Plausible interactions between multimorbidity and baseline factors were also examined.

The independent association between the nine chronic diseases and mortality was determined by replacing multimorbidity with nine binary variables representing each of the nine chronic diseases in the Cox proportional hazards models. Adjusted hazard ratios for the nine chronic diseases were also estimated, along with plausible interactions between chronic diseases. To account for survey design, non-response and refusal to link, design weights provided by Statistics Canada were used in all the analyses. Bootstrap weights (with 500 iterations) were applied to estimate variances and 95% confidence intervals as suggested by Statistics Canada to adjust for the complex survey design.^26^ Stata/MP 16 (StataCorp LP, College Station, TX) and SAS 9.4 (SAS Institute Inc., Gary, North Carolina, USA) were used for statistical analyses.

## Results

The follow-up characteristics for each CHHS survey is shown in Table 1. Number of deaths decreased from CCHS 2003/2004 to CCHS 2014 which was expected since subjects interviewed in initial surveys were older and had a higher risk of death during the follow-up than those interviewed in the latter surveys. In total, 434,885 subjects were followed up and during the follow-up, 64,370 died and 370,155 were censored.

**Table 1.** Description of follow-up from the linkage of the Canadian Community Health Surveys (2003–2014) with the Canadian Vital Statistics—Death database.*

Year of entry	Number of people included in analysis	Number of deaths	Total survival time (days)	Proportion of deaths (%)	Number censored	Total survival time (days)	Proportion of censored (%)
2003/2004	71650	17505	49981490	24.43	54145	287048205	75.57
2005/2006	73085	15125	37330265	20.70	57960	264652080	79.30
2007	38725	6580	13771840	16.99	32145	123540730	83.01
2008	37495	5720	10724955	15.26	31775	110604045	84.74
2009	35190	4675	7974535	13.29	30515	95018670	86.71
2010	34935	4255	6347420	12.18	30680	84360925	87.82
2011	35400	3535	4584620	9.99	31865	75935835	90.01
2012	34995	2940	3306575	8.40	32055	64703080	91.60
2013	36930	2600	2341075	7.04	34330	56825425	92.96
2014	36480	1795	1276625	4.92	34685	44855700	95.08
Total	434885	64730	137639400		370155	1207544695	

* Figures in the table were rounded off using base-five rounding rules.

### The relationship between multimorbidity and all-cause mortality

Overall incidence of death and unadjusted risk ratios for all cause mortality are shown in Table 2 for the nine chronic diseases and multimorbidity. The overall risk of death was significantly greater for subjects with multimorbidity in comparison to those without multimorbidity (HR = 6.15; 95 % CI: 5.91, 6.41; *p* < 0.001). Each chronic disease was independently associated with an increased risk of death, with the lowest and highest risk being in subjects with mood disorder and stroke, respectively.

**Table 2.** The distribution of incidence of death and unadjusted hazard ratios for the nine chronic diseases and multimorbidity.

Disease group	Incidence (%)	Hazard ratio (95% CI)	p-value
Multimorbidity			
No	0.04	1.00	
Yes	0.20	6.15 (5.91, 6.41)	<0.001
Mood Disorder			
No	0.09	1.00	
Yes	0.10	1.21 (1.15, 1.28)	<0.001
Asthma			
No	0.09	1.00	
Yes	0.11	1.29 (1.23, 1.36)	<0.001
Arthritis			
No	0.07	1.00	
Yes	0.16	2.41 (2.35, 2.48)	<0.001
High Blood Pressure			
No	0.07	1.00	
Yes	0.16	2.65 (2.57, 2.73)	<0.001
Diabetes			
No	0.08	1.00	
Yes	0.20	2.97 (2.86, 3.08)	<0.001
COPD			
No	0.09	1.00	
Yes	0.25	4.22 (4.01, 4.44)	<0.001
Heart Disease			
No	0.08	1.00	
Yes	0.28	4.34 (4.19, 4.50)	<0.001
Cancer			
No	0.08	1.00	
Yes	0.31	4.68 (4.43, 4.94)	<0.001
Stroke			
No	0.09	1.00	
Yes	0.36	5.24 (4.94, 5.56)	<0.001

**Figure 1.**
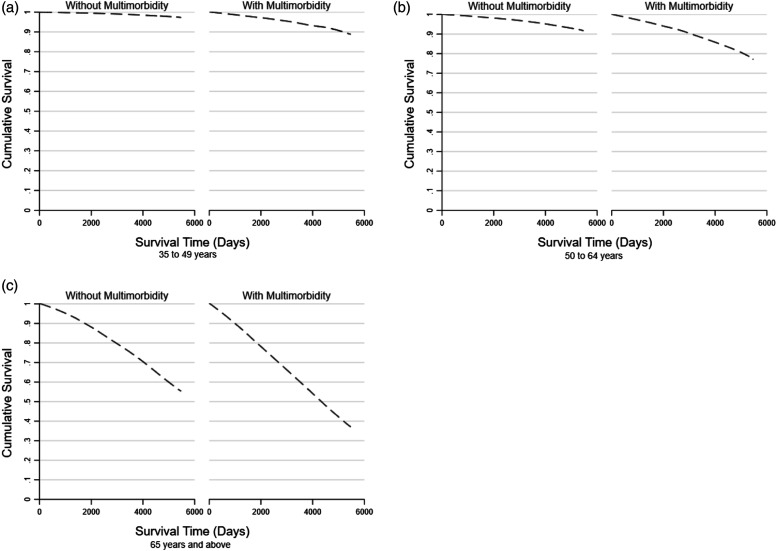
Smoothed Kaplan-Meier curves of cumulative survival probability for subjects with and without multimorbidity by age group.

As shown in Figure 1, there was a rapid decrease in the survival rates in older age groups in comparison to the younger age groups and the survival rates for subjects with multimorbidity was lower than that for subjects without multimorbidity in all the three age groups. The results from stratified analysis by age groups are shown in Table 3. The hazard ratio for mortality for subjects with multimorbidity in comparison to those without decreased as age increased from the youngest age group to the oldest age group (35 to 54 years: HR = 4.77, 95% CI: 3.94, 5.77; 55 to 64 years: HR = 3.16, 95% CI: 2.87, 3.48; 65 years and above: HR = 1.52; 95% CI:1.74,1.90).

**Table 3.** Adjusted hazard ratios of multimorbidity and baseline factors without interactions for the three age groups: Results from the multiple logistic regression.*

Disease group/baseline factors	35 to 49 years hazard ratio (95% CI)	50 to 64 years hazard ratio (95% CI)	65 years and above hazard ratio (95% CI)
Multimorbidity			
No	1.00	1.00	1.00
Yes	3.77 (3.04, 4.67)	2.64 (2.36, 2.95)	1.71 (1.63, 1.80)
Sex			
Female	1.00	1.00	1.00
Male	1.83 (1.50, 2.23)	1.81 (1.65, 1.99)	1.77 (1.68, 1.85)
Racial Background			
Non-white			1.00
White	-	-	1.97 (1.71, 2.26)
Body weight			
Under weight	1.00	1.00	1.00
Normal weight	0.42 (0.27, 0.65)	0.53 (0.41, 0.70)	0.51 (0.45, 0.58)
Overweight	0.33 (0.21, 0.52)	0.50 (0.38, 0.67)	0.36 (0.31, 0.40)
Obese	0.47 (0.30, 0.74)	0.57 (0.43, 0.75)	0.35 (0.31, 0.40)
Marital status			
Single	1.00	1.00	1.00
Widowed/Divorced/Separated	0.81 (0.63, 1.04)	1.07 (0.91, 1.26)	1.21 (1.11, 1.32)
Common-law/Married	0.66 (0.53, 0.82)	0.70 (0.61, 0.80)	0.75 (0.69, 0.82)
Province of residence			
British Columbia			1.00
Prairies			0.90 (0.84, 0.96)
Quebec	-	-	0.76 (0.70, 0.82)
Ontario			0.81 (0.76, 0.87)
Atlantic			0.80 (0.75, 0.87)
Territories			0.83 (0.64, 1.08)
Household income			
less than $39,999	1.00	1.00	1.00
$40,000 to $59,999	0.78 (0.60, 1.02)	0.84 (0.70, 1.00)	0.84 (0.79, 0.90)
$60,000 to $79,999	0.78 (0.56, 1.07)	0.74 (0.63, 0.86)	0.83 (0.75, 0.91)
$80,000 or more	0.61 (0.48, 0.79)	0.66 (0.59, 0.74)	0.94 (0.89, 0.98)
Highest household education			
Less than secondary school graduation	1.00	1.00	1.00
Secondary school graduation, no post-secondary education	0.91 (0.68, 1.21)	0.72 (0.64, 0.80)	0.89 (0.84, 0.94)
Some post-secondary education	0.68 (0.50, 0.92)	0.82 (0.69, 0.97)	0.86 (0.78, 0.95)
Post-secondary and above	0.67 (0.55, 0.82)	0.81 (0.73. 0.89)	0.77 (0.73, 0.80)
Smoking status			
Non-Smoker	1.00	1.00	1.00
Smoker	1.79 (1.50, 2.14)	1.99 (1.81, 2.18)	1.30 (1.23, 1.38)
Alcohol			
No	1.00	1.00	1.00
Moderate	0.82 (0.66, 1.03)	0.72 (0.63, 0.82)	0.72 (0.69, 0.76)
Heavy	1.28 (0.92, 1.78)	0.85 (0.73, 1.00)	0.82 (0.76, 0.88)
Physical activity			
Inactive		1.00	1.00
Moderately	-	0.79 (0.71, 0.88)	0.62 (0.59, 0.65)
Active		0.73 (0.64, 0.82)	0.53 (0.49, 0.56)
Stress			
Not and not very stressful		1.00	1.00
Somewhat	-	0.82 (0.75, 0.91)	0.93 (0.90, 0.97)
Extreme		0.88 (0.74, 1.05)	0.96 (0.81, 1.14)

* Only significant baseline variables were kept in the proportional hazards models

Age-specific incidence of death and unadjusted hazard ratios for the baseline factors are also shown in Supplementary Table S1. Male subjects had a higher incidence of deaths than female subjects in all the three age groups. Whites, smokers, and subjects with extreme stress had an increased risk of death in all the three age groups. In the three age groups, subjects who were married or in a common-law relationship had a lower risk of death than those who were single. In comparison to underweight categories, other weight categories had a decreased risk of death, and similarly increasing household income, higher education and regular physical activity was associated with a decreased risk of death in all three age groups. Moderate and heavy drinkers had a significantly lower risk of death in comparison to non-drinkers in the middle and older age groups but the observed lower risk for heavy drinkers in the youngest age group was not statistically significant. The results from the final model using the multivariable logistic regression analysis are shown in Table 3 for the three age groups. All the baseline factors were significant in the final model for the oldest age group whereas only some of the baseline factors were significant in the other age groups. In all three age groups, multimorbidity was associated with an increased risk of mortality after adjusting for significant baseline factors. Adjusted hazard ratios for multimorbidity decreased from the youngest age group (HR = 3.77; 95% CI: 3.04, 4.67) to the oldest age group (HR = 1.71; 95% CI: 1.63, 1.80).

### The independent and interaction effects of chronic diseases on mortality

In a further multivariable analysis, binary variables indicating each of the nine chronic diseases individually were considered simultaneously instead of the single binary variable corresponding to multimorbidity in the proportional hazards model for the three age groups. As show in Table 4, after adjusting for the significant baseline factors, of the nine chronic diseases, asthma and heart disease were not significantly associated with the risk of death in the youngest age group. Asthma, arthritis, and mood disorder were not significantly associated with the risk of death in the middle age group. Asthma and arthritis were not significantly associated with risk of death in the oldest age group. As inferred from these results, asthma was not associated with death in any of the three age groups after adjusting for other chronic diseases and significant baseline factors. Subjects with cancer had the highest risk of death after adjusting for the other chronic diseases and significant baseline factors in the youngest and middle age groups. In the oldest age group, subjects with COPD and cancer had the highest risk of death after adjusting other chronic diseases and significant baseline factors. It is also worth noting that mood disorders had a marginally significant protective effect of death in the oldest age group (HR = 0.92; 95% CI: 0.86, 0.99).

**Table 4.** Adjusted hazard ratios of nine chronic diseases without interactions by age groups: Results from the multiple logistic regression.*

Disease group/baseline factors	35 to 49 years hazard ratio (95% CI)	50 to 64 years hazard ratio (95% CI)	65 years and above hazard ratio (95% CI)
Cancer			
No	1.00	1.00	1.00
Yes	10.72 (7.61, 15.09)	4.23 (3.73, 4.79)	1.75 (1.63, 1.87)
COPD			
No	1.00	1.00	1.00
Yes	2.61 (1.55, 4.40)	1.75 (1.53, 2.01)	1.75 (1.64, 1.88)
Diabetes			
No	1.00	1.00	1.00
Yes	1.94 (1.54, 2.44)	1.76 (1.59, 1.96)	1.39 (1.33, 1.45)
Stroke			
No	1.00	1.00	1.00
Yes	1.65 (1.04, 2.62)	1.61 (1.32, 1.97)	1.49 (1.39, 1.60)
High Blood Pressure			
No	1.00	1.00	1.00
Yes	1.48 (1.23, 1.78)	1.26 (1.15,1.37)	1.06 (1.02, 1.10)
Heart Disease			
No	1.00	1.00	1.00
Yes	1.32 (0.99, 1.77)	1.65 (1.47, 1.85)	1.53 (1.47, 1.59)
Mood Disorder			
No	1.00	1.00	1.00
Yes	1.38 (1.10, 1.73)	1.09 (0.97, 1.22)	0.92 (0.86, 0.99)
Arthritis			
No	1.00	1.00	1.00
Yes	1.27 (1.05, 1.52)	1.04 (0.97, 1.22)	1.02 (0.99, 1.06)
Asthma			
No	1.00	1.00	1.00
Yes	1.10 (0.84, 1.43)	1.09 (0.96, 1.23)	1.02 (0.96, 1.08)

* Adjusted for the baseline factors in Table 4 for each age.

Significance of plausible interactions between the nine chronic diseases and baseline factors were examined in the proportional hazards models after adjusting for the significant baseline factors in the three age groups. As shown in the supplemantry Table S2, The interaction between asthma and mood disorder was significant in the oldest age group, with an increased risk of death among subjects with asthma in the absence of mood disorder (HR = 1.19; 95 % CI: 1.04, 1.36), and a non-significant decreased risk in the presence of mood disorder (HR = 0.91; 95 % CI:0.72, 1.15) in comparison to subjects without asthma or mood disorder. An interaction between COPD and diabetes was also significant in the oldest age group with there being an increased risk of death among subjects with COPD alone (HR = 1.93; 95% CI:1.66, 2.24), but notably a significantly greater risk of death in the presence of diabetes (HR = 2.59; 95% CI:2.01, 3.34) in comparison to subjects without COPD or diabetes.

## Discussion

In this retrospective cohort study, we found that multimorbidity was positively associated with all-cause mortality among younger, middle-age and older Canadians. The effect of multimorbidity on the risk of deaths decreased from the youngest age group to the oldest age group. Subjects with cancer had the highest risk of death in the three age groups after controlling for other chronic diseases. Significant interactions between asthma and mood disorder and COPD and diabetes were observed in the association with the risk of death in the oldest age group.

Evidence of a proportional decreasing risk of mortality with aging in people with multimorbidity has been reported in other studies.^27,28^ A retrospective cohort study based on the Ontario population in Canada showed that the mortality rate ratios of all-cause mortality increased from the early and middle age groups (18 to 24 years; rate ratio = 9.96; 95% CI: 7.18, 18.34 ) to older ages (80+ years; rate ratio = 1.97, 95% CI:1.94, 2.04) after adjusting for potential confounders. These trends were observed when using three or more chronic conditions to define multimorbidity.^7^ Similar results were observed in a study based on UK biobank data which reported that the impact of multimorbidity on all-cause mortality was greater in the middle age group than the older age group , particularly in males.^14^ In a prospective cohort study of older adults from central Italy it was found that disability, rather than multimorbidity, had a greater impact on mortality in the elderly aged over 80 years and above.^29^ However, the biological mechanism for the effect of aging on multimorbidity related mortality remained unknown. It was reported that a survival effect could be a possible explanation, with the hypothesis being that life-threatening multimobidities lead to premature death.^30^

In this study, significant interactions between asthma and mood disorder and diabetes and COPD, with risk of death were found in subjects aged 45 to 64 years. The coexistence of mood disorder in patients with asthma has previously been reported to be associated with increased mortality.^31^ For example, a cohort study of veterans with asthma in the United States found that among veterans with asthma aged 46 to 64 years old, coexisting mental disorder was associated with increased all-cause mortality in comparison to those with asthma alone, after adjusting the confounding effects.^32^ Although there have been only a few studies focusing on the association of comorbidity with asthma and mental illness with mortality, one study has reported that patients with co-existing asthma and depression had a greater number of emergency room visits due to asthma-related complications.^33^ However, in this study, there was a non-significant protective effect of mood disorder on all-cause mortality among subjects with asthma. The joint effect of diabetes and COPD on mortality has also been investigated in other studies. Similar to the finding in this study, a multicenter cohort study of COPD patients found that those with diabetes had an increased risk of death in comparison to those without diabetes. (HR = 1.7; 95% CI:1.2, 2.4).^34^ The underlying biological mechanism is still unknown and possibly multifactorial. It has been suggested that the increased glucose concentration in airway secretions in diabetes may result an increased risk of infective exacerbations and consequently in more severe symptoms related to COPD, and an increased risk of mortality.^35^

This study has both strengths and limitations. The majority of the previous studies on multimorbidity and mortality were based on provincial databases, whereas this study was based on a nationwide health survey representing the entire Canadian population. The large sample size potentially reduced selection bias and sampling errors. To the best of the authors’ knowledge, it is the first study using a linked database of CCHS-CVSD data to examine the relationship between multimorbidity and mortality among the Canadian population. Recognizing multimorbidity associated mortality is multifactorial, this study included a wide range of factors in the analysis to adjust for possible confounding. Furthermore, this study examined the joint effects between chronic diseases among subjects with multimorbidity. These results may guide the healthcare professional to intervene, provide appropriate treatment and manage comorbidity. There are some limitations in the study as well. Unlike other studies where chronic diseases were weighted according to the severity of the diseases and effects of disease-related functional impairments,^36^ the effect of each chronic disease was weighted equally in this study. Additionally, the presence of chronic diseases was identified by a self-report of a health professional diagnosis, which could lead to potential underestimation of multimorbidity. Although the proportional hazard assumption was violated in people aged 65 years and above,an attempt was made to include an interaction variable between multimorbidity and time. However, there were convergence issues in the estimation of parameters in the Cox's model which was related to the large sample size and the results from the model with the interaction were not reported.

## Conclusion

The association between multimorbidity and mortality was significant across all age groups with decreasing proportional risk from the younger to older age groups. In this study, diabetes had an increased risk of mortality among subjects with COPD. Given that multimorbidity had a proportionately greater effect on mortality among younger age groups than older age group in this study, prevention goals should target younger and middle-aged subjects in order to reduce excess mortality associated with multimorbidity. Additional information on the causes of death is required to delineate the mortality associated with multimorbidity from other causes and establish causal relationships between multimorbidity and mortality.

## Supplemental Material

Supplemental Material - Mortality among Canadian population with multimorbidity: A retrospective cohort study Click here for additional data file. Supplemental Material for Mortality among Canadian population with multimorbidity: A retrospective cohort study by Xiang Xiao, Msc, Jeremy Beach, MD, Ambikaipakan Senthilselvan, PhD in Journal of Multimorbidity and Comorbidity
