# Supplementary material for: Mortality among Canadian population with multimorbidity: A retrospective cohort study
Source: J Multimorb Comorb. 2023 Feb 17;13:26335565231157626. doi: 10.1177/26335565231157626 (PMC9940159; doi:10.1177/26335565231157626)
Supplement: Supplemental Material - Mortality among Canadian population with multimorbidity: A retrospective cohort study [file sj-pdf-1-cob-10.1177_26335565231157626.pdf]

SUPPLEMENTARY MATERIALS

Table S1. Incidence of death and unadjusted hazard ratios for multimorbidity and baseline factors for the three age groups.

	35 to 49 years		50 to 64 years		65 years and above	
Disease group/baseline factors	Incidence	Hazard ratio (95% CI)	Incidence	Hazard ratio (95% CI)	Incidence	Hazard ratio (95% CI)
Multimorbidity						
No	0.01	1.00	0.03	1.00	0.20	1.00
Yes	0.05	4.77 (3.94, 5.77)	0.10	3.16 (2.87, 3.48)	0.32	1.52 (1.74,1.90)
Sex						
Female	0.01	1.00	0.04	1.00	0.25	1.00
Male	0.02	1.56 (1.35, 1.81)	0.07	1.56 (1.45, 1.67)	0.29	1.28 (1.22,1.32)
Racial Background						
Non-white	0.01	1.00	0.03	1.00	0.17	1.00
White	0.02	1.44 (1.07, 1.94)	0.06	1.81 (1.46, 2.25)	0.28	1.66 (1.48, 1.86)
Body weight						
Under weight	0.04	1.00	0.11	1.00	0.46	1.00
Normal weight	0.02	0.42 (0.30, 0.59)	0.05	0.42 (0.34, 0.51)	0.28	0.51 (0.46, 0.56)
Overweight	0.01	0.37 (0.26, 0.53)	0.05	0.41 (0.33, 0.50)	0.23	0.40 (0.37, 0.44)
Obese	0.02	0.62 (0.44, 0.88)	0.07	0.60 (0.49, 0.74)	0.29	0.59 (0.53, 0.65)
Marital status						
Single	0.03	1.00	0.08	1.00	0.28	1.00
Widowed/Divorced/Separated	0.02	0.81 (0.65, 1.00)	0.08	0.99 (0.87, 1.13)	0.34	1.17 (1.09, 1.26)
Common-law/Married	0.01	0.40 (0.34, 0.47)	0.05	0.55 (0.49, 0.61)	0.23	0.74 (0.69, 0.80)
Province of residence						
British Columbia	0.02	1.00	0.05	1.00	0.28	1.00
Prairies	0.02	0.82 (0.64, 1.05)	0.06	1.15 (1.02, 1.31)	0.28	1.02 (0.97, 1.07)
Quebec	0.01	0.69 (0.52, 0.89)	0.06	1.12 (0.99, 1.26)	0.25	0.89 (0.84, 0.94)
Ontario	0.02	0.80 (0.64, 1.00)	0.05	1.04 (0.93, 1.17)	0.26	0.92 (0.87, 0.97)
Atlantic	0.02	0.88 (0.70, 1.13)	0.07	1.36 (1.21, 1.53)	0.29	1.08 (1.02, 1.14)
Territories	0.02	1.06 (0.72, 1.55)	0.07	1.40 (1.12, 1.75)	0.30	1.16 (0.99, 1.35)
Household income						

less than $39,999	0.03	1.00	0.09	1.00	0.30	1.00
$40,000 to $59,999	0.02	0.62 (0.59, 0.76)	0.06	0.60 (0.53, 0.69)	0.21	0.73 (0.69, 0.76)
$60,000 to $79,999	0.02	0.45 (0.38, 0.62)	0.04	0.45 (0.40, 0.51)	0.18	0.65 (0.61, 0.70)
$80,000 or more	0.01	0.37 (0.31, 0.43)	0.04	0.41 (0.37, 0.44)	0.29	0.82 (0.79, 0.85)
Highest household education						
Less than secondary school graduation	0.04	1.00	0.09	1.00	0.34	1.00
Secondary school graduation, no post-secondary education	0.02	0.60 (0.48, 0.75)	0.05	0.54 (0.49, 0.59)	0.25	0.78 (0.74, 0.81)
Some post-secondary education	0.02	0.55 (0.42, 0.70)	0.07	0.70 (0.59, 0.83)	0.28	0.81 (0.74, 0.89)
Post-secondary and above	0.01	0.38 (0.33, 0.45)	0.05	0.53 (0.49, 0.57)	0.21	0.66 (0.63, 0.68)
Smoking status						
Non-Smoker	0.01	1.00	0.04	1.00	0.26	1.00
Smoker	0.02	3.72 (2.38, 3.12)	0.10	2.55 (2.27, 2.74)	0.37	1.56 (1.50, 1.63)
Alcohol						
No	0.02	1.00	0.08	1.00	0.34	1.00
Moderate	0.01	0.68 (0.57,0.82)	0.05	0.57 (0.52, 0.63)	0.23	0.63 (0.61, 0.65)
Heavy	0.03	1.28 (0.99, 1.66)	0.06	0.71 (0.62, 0.81)	0.26	0.70 (0.67, 0.74)
Physical activity						
Inactive	0.02	1.00	0.07	1.00	0.31	1.00
Moderate	0.01	0.74 (0.62, 0.88)	0.04	0.64 (0.59, 0.70)	0.19	0.58 (0.56, 0.61)
Active	0.01	0.73 (0.61, 0.87)	0.04	0.59 (0.54, 0.65)	0.16	0.50 (0.48, 0.53)
Stress						
Not and not very stressful	0.02	1.00	0.06	1.00	0.27	1.00
Somewhat	0.01	0.88 (0.76, 1.03)	0.05	0.92 (0.85, 0.99)	0.26	1.01 (0.97, 1.04)
Extreme	0.03	1.61(1.18, 2.22)	0.07	1.31 (1.14, 1.51)	0.34	1.29 (1.22, 1.58)
Year of entry						
2003/2004	0.04	1.00	0.13	1.00	0.54	1.00
2005/2006	0.03	0.90 (0.76, 1.06)	0.01	0.95 (0.88, 1.04)	0.46	0.98 (0.94, 1.03)
2007	0.03	1.34 (1.10, 1.67)	0.01	0.88 (0.79, 0.99)	0.36	0.94 (0.88, 0.99)
2008	0.01	0.76 (0.60, 0.96)	0.05	0.95 (0.84, 1.07)	0.32	0.92 (0.87, 0.98)
2009	0.01	0.76 (0.58, 1.27)	0.05	0.78 (0.67, 0.91)	0.28	0.91 (0.85, 0.96)

2010	0.01	0.90 (0.64, 1.26)	0.04	0.83 (0.72, 0.95)	0.25	0.93 (0.87. 0.99)
2011	0.01	0.89 (0.62, 1.37)	0.04	0.97 (0.77, 1.21)	0.21	0.94 (0.87, 1.03)
2012	0.01	0.92 (0.63, 1.87)	0.03	0.84 (0.69, 1.04)	0.17	0.90 (0.83, 0.97)
2013	0.01	1.09 (0.61, 2.79)	0.02	0.81 (0.67, 0.95)	0.13	0.90 (0.82, 0.98)
2014	0.01	1.31 (0.61, 1.14)	0.02	0.89 (0.69, 1.15)	0.09	0.81 (0.74, 0.89)

Table S2 Adjusted hazard ratios for significant interactions between comorbidities from the multivariable proportional hazards model for 50 to 64 years.

Disease groups		Hazard ratio (95% CI)	p-value
Asthma	Mood disorder		
No	No	1.00	
Yes	No	1.19 (1.04, 1.36)	0.01
No	Yes	1.17 (1.04, 1.33)	0.01
Yes	Yes	0.91 (0.72, 1.15)	0.44
Diabetes	COPD		
No	No	1.00	
Yes	No	1.83 (1.64, 2.04)	<0.001
No	Yes	1.93 (1.66, 2.24)	<0.001
Yes	Yes	2.59 (2.01, 3.34)	<0.001
